# Assessment of Scattered Dose to the Eye in Dentistry: A Systematic Review

**DOI:** 10.7759/cureus.43113

**Published:** 2023-08-08

**Authors:** Mawya Khafaji, Ghaidaa H Albadawi

**Affiliations:** 1 Radiology, King Abdulaziz University Faculty of Medicine, Jeddah, SAU; 2 Dentistry, King Abdulaziz University, Jeddah, SAU

**Keywords:** radiation dosimetry, cbct, scatter radiation, eye, dental treatment

## Abstract

Cone-beam computed tomography (CBCT) is a tool for dental imaging of impactions, maxillofacial discrepancies, facial trauma, and tumors. In addition, It is used in treatment planning for dental implants, orthognathic surgery, and general maxillofacial surgery. There are no standardized methods for utilizing CBCT dosimetry, and there is no consensus among dental and medical physics health professionals regarding dental CBCT imaging procedures. The eyes and thyroid glands are radiosensitive organs that lie outside the primary beam but receive a significant amount of radiation due to scattered radiation. This study aimed to assess the dose to eye lens in patients imaged using CBCT. This review aims to evaluate the scattered doses to the eye from CBCT among adult patients seeking dental treatment. The search included published articles in the Web of Science, PubMed (MeSH and Web PubMed), Medline, and Google Scholar databases using the appropriate keywords from January 2010 to July 2022. The inclusion criteria were based on the method of dose measurement (phantom studies using Optically stimulated luminescence (OSL) and Thermoluminescent dosimeter (TLD), language, and type of protocol used. A literature search was performed using the Preferred Reporting Items for Systematic Reviews and Meta-Analyses checklist and flow chart. Out of 653 articles identified, 5 met the inclusion criteria. The results show that the scattered radiation dose ranged between 0.103 mSv and 8.3 mSv. This variation exists due to the difference in the field of vision (FOV), phantom exposure, dosimeters used, degree of rotation in the protocol, and finally, the scanner used. The scattered dose to the eye from CBCT is higher than the background radiation, with huge variability in the range of the dose measured. Clear guidelines for utilizing CBCT should be implemented, and dose reference levels should be established for benchmarking and optimization in practice.

## Introduction and background

The use of CBCT cone-beam computed tomography increased dramatically in the last decade in dental practice to assess maxillofacial structures for diagnostic, treatment planning, and follow-up purposes. Almost 4 million CBCT examinations are estimated to be performed annually in the United States of America (USA) alone [1,2]. CBCT is a new modality consisting of a cone-shaped beam that rotates around the patient's head to acquire raw two-dimensional images reconstructed from several projections to form a three-dimensional (3D) volume and is associated with the highest patient dose [3,4]. The cumulative doses from CBCT machines can range from 5 to 1,073 micro Sieverts (µSv) [5]. Ionizing radiation used in CBCT is associated with an increased risk of developing leukemia and other cancers in any patient's life span [6].

The effective dose is the recommended parameter to express the risk from radiation exposure by ICRP. The International Commission on Radiological Protection (considers the biological effect on radiosensitive tissues/organs using weighting factors depending on the degree of organ sensitivity [7]. Computed tomography (CT) Dose Index or Dose Area Product are exposure indicators for CT that can be used for calculating the CBCT dose [3]. In addition, field of view (FOV), collimation, exposure parameters, and patient age and size affect the amount of absorbed dose received during CBCT examinations. According to the Food and Drug Administration, "radiation doses from dental CBCT exams are generally lower than that from other CT exams, and dental CBCT exams typically deliver more radiation than conventional dental X-ray exams" [8].

The effective dose from CBCT was reported to be between 46 and 1073 Sv in adult phantom studies [9,10]. Although the guidelines suggest that the dose from the CBCT modality is equivalent to doses from 2 to 10 panoramic radiographs, it has been reported that the dose can range from 2 to 200 panoramic radiographs [11]. This big variation emphasizes the need for practice standardization and justification of use [12].

In addition to the exposure from direct beams, scattered radiation is of concern, especially in head imaging, where the eye lens could receive an unnecessary dose. [13] The ICRP has dropped the yearly occupational eye dose considerably from 150 mSv to 20 mSv after epidemiological evidence proved damage to the eye lens with radiation exposure. This has changed the threshold for this sensitive organ compared to the past [14]. Scattered radiation associated with CBCT can be reduced using proper techniques, including collimation, protocol, and FOV position [15]. Alwasiah et al. in 2021 reported that the mean dose absorbed by the eyes was 33.6 mGy. Moreover, they have indicated that these numbers are alarming as damage could be induced in the eyes due to radiation doses "as low as 0.2 and 0.5 Gy" [16].

Factors such as FOV and the location of radiosensitive organs impact the patient radiation dose; using a larger FOV exposes more tissue to radiation and results in more scattering to adjacent areas. In addition, the FOV position (depending on the protocol) also affected the dose. A volume-dose model was proposed by Pauwel et al. 2014 [17] using various FOVs and FOV positions to optimize patient doses and reduce scatter to radiosensitive organs. Their results showed a significant dose reduction (64-69%) when using the same FOV for the mandible instead of the maxillofacial region. A dose reduction of more than 30% was achieved in the mandibular position.

When the FOV from 17x 2 to 14 × 5 cm. They also observed a higher scattered dose to the thyroid when mandibular scans were performed. Most important, "FOV should not be positioned inferiorly to achieve a reduction of the eye lens dose" [18] because this, in turn, could increase the thyroid dose. Therefore, reducing the eye lens dose is achievable only by using a smaller FOV or decreasing the mAs used.

## Review

Methodology

Study Identification

The PECO was used to formulate the search question; Population (Adult patient undergoing dental treatment); Exposure (CBCT); Comparison (X); Outcome (scattered dose to the eye lens). 

The search terms are: (adult dental procedure) AND (dose scatter CBCT) AND (scattered dose to the eye CBCT radiation). An electronic search was formed on July 2022 using keywords and MeSH terms in PubMed, Medline, WebMD, and google scholar databases with a restriction of an English language and year of publication since 2010. 

**Figure 1:**
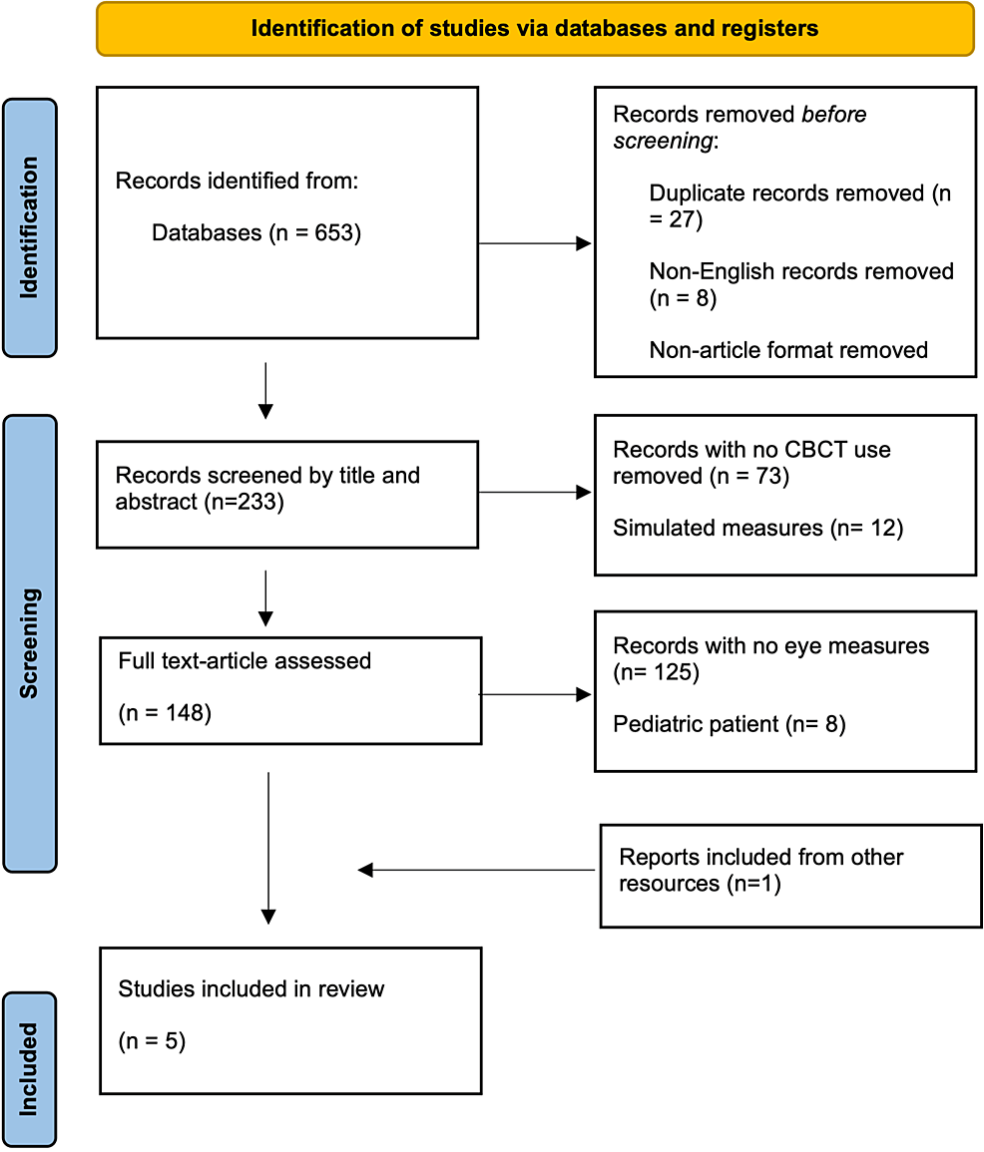
PRISMA 2020 flow diagram for new systematic reviews.

Articles titles and abstracts were evaluated based on inclusion and exclusion criteria (figure 1), and the full texts of the selected articles were downloaded for scrutiny. Articles meeting all the criteria were selected for data extraction. Each of these steps was performed by two reviewers who excluded articles on which they disagreed (Table 1).

Data Extraction

Data extraction was performed using a self-designed pre-piloted data extraction form, which was pilot tested in a sample of four studies. 

Elements extracted were: (a) author (first author); (b) year; (c) country (d) aim of the study; (e) amount of TLDs; (f) type of phantom; (g) type of device; (h) field of view FOV; (i) rotation; (j) unit; (k) type of dose. 

Inclusion criteria: Relevant articles were screened by their titles and abstracts after removing duplicates. Studies were included if they addressed the measurement of radiation from CBCT on dental structures regardless of the type of device or phantom used. In addition, adult protocol, dental treatment, phantom head, absorbed dose readings, and effective dose readings. 

Exclusion criteria: For any study involving pediatric patients, no reading of the dose to the eye, usage of collimations, non-phantom head, non-English-language papers, non-articles, and simulated measurements. 

According to Preferred Reporting Items for Systematic Reviews and Meta-Analyses (PRISMA), any disagreement on selecting the study was evaluated by another examiner [19].

Results

According to the PRISMA, our search included more than 650 records, which were then analyzed and resulted in 5 articles (Fig 1).

R. Prins (2011)

In New York, USA, a study was conducted to investigate the effect of wearing leaded eyeglasses during dental CBCT imaging on the radiation dose absorbed by the eye. Three anthropomorphic phantoms were used: male (Alderson radiation therapy phantom), female (CIRS (Computerized Imaging Reference System) phantom), and juvenile male (CIRS phantom). The scans were performed using an Illumina CBCT machine. This study reported eye measurements with collimation that were not included in our study. Eye doses were measured in two situations: with/without (0.051±0.002 and 0.133±0.002), respectively. Leading glasses worn by adult patients during CBCT scans may reduce radiation dose to the eye lens by 67% [20]. 

AD Goren (2013)

This study was performed in New York, USA., to investigate the dose to the lens and thyroid after wearing leaded glasses and a thyroid shield using an anthropomorphic female phantom and a commercially available CBCT with five exposure settings. A 61% reduction was observed after exposure to the eye lenses, from 0.396 to 0.153 mGy [21]. 

R Pauwels (2014)

An experimental study was conducted in the European Union and the United Kingdom to quantify the effect of the FOV and angle of rotation on the radiation dose in dental CBCT. An ART (Alderson Radiation Therapy)phantom equipped with a TLD ship (TLD-100) was used. The effective doses to the eye were 54-303 mSv at 360° and 95-6,861 mGy at 180°. This study was restricted to effective dose measurements [17]. 

E Hofmann (2014)

A study in Germany was conducted to evaluate image quality and dose exposure of different (CBCT) and (MSCT) Multislice spiral computed tomography scanners: 3D Accuitomo 170 (J. Morita Inc., Japan), 3D eXam (KaVo Dental GmbH, Germany), Pax Zenith 3D, Pax Reve 3D, and Picasso Trio 3D (E-Woo) using TLD. Using an anthropomorphic head and neck scanner (Alderson RANDO). Doses absorbed by the eye lenses from the five scanners are addressed in (table 2) where the 3D Accuitomo 170 had an extremely high dose of 8.30 mSv on the other hand, the other four scans showed comparable scan heights, displayed enormous differences that did not exceed 2.38 mSv regarding the dose measured at the TLD around the eye lens [22]. 

S Mutalik (2020)

Another study was performed in Connecticut, USA, to compare the effective doses of 360° versus 180° rotations with different FOVs in CBCT. 3D Accuitomo 170 CBCT Scanner was used with a RANDO anthropometric phantom. Light nanodot dosimeters were used to measure absorbed radiation dose. Both absorbed and effective doses were substantially lower for the 180° rotation scans (P < 0.01). The doses were generally lower for small FOVs than those for large FOVs. The average doses within the eye regions (mGy) were measured 15 times for each 360° and 180° rotation [23]. 

**Table 1:** List of articles used in this systematic review with their dose type, dosimeter used, and eye measures. CBCT: Cone-beam computed tomography; OSL: Optically stimulated luminescence; TLD: Thermoluminescent dosimeter; ART: Alderson Radiation Therapy

Ref. no.	Year	Country	Rotation		Dose type		Dosimeter type	CBCT type/s	Phantom type	No. of eye measures	
			360°	180°	Effective	Absorbed				Average	Two separate lenses
[20]	2011	USA	-	-		√	OSLs	Iluma CBCT	-Adult female - Adult male	√	
[21]	2013	USA	-	-		√	OSLs	i-CAT Platinum	Adult female		√
[17]	2014	UK	√	√	√		TLDs	3D Accuitomo 170 CBCT	ART		√
[22]	2014	Germany	-	-		√	TLDs	- 3D Accuitomo 170 - 3D eXam - Pax Zenith 3D - Pax Reve 3D - Picasso Trio 3D	Rando	√	
[23]	2020	USA	√	√	√		OSLs	3-D Accuitomo 170	Rando		√

**Table 2:** Summary of each study with their eye measures according to FOV, Rotation degree, and device used. FOV: field of vision

Paper	Rotation degree	FOV	Effective dose in mGy					
R Pauwels (2014) [17]	360°	40 x 40 Upper canine	0.174					
		40 x 40 Lower molar	0.146					
		60 x 60 Upper frontal	0.608					
		60 x 60 Lower molar	0.255					
		140 x 50 Upper jaw	0.681					
		140 x 50 Lower jaw	0.203					
		80 x 80 Both jaws	0.692					
		100 x 100 Both jaws	5.308					
		170 x 120 Maxillofacial	6.861					
	180°	40 x 40 Upper canine	0.095					
		60 x 60 Upper frontal	0.288					
		60 x 60 Lower molar	0.103					
		80 x 80 Both jaws	0.292					
		170 x 120 Maxillofacial	2.284					
Absorbed doses (mGy)								
			Lens of right eye			Lens of left eye		
S Mutalik (2020) [23]	360°	170 x 120	8.1			8.0		
		140 x100	1.5			1.0		
		100 x 100	1.6			1.7		
		80 x 80	0.7			1.8		
		60 x 60 Anterior maxilla	0.5			0.4		
		60 x 60 Right maxillary molar	0.6			0.7		
		60 x 60 Right mandibular molar	0.7			1.2		
		40 x 40 Anterior maxilla	0.7			0.6		
		40 x 40 Right maxillary molar	0.2			0.2		
		40 x 40 Right mandibular molar	0.5			0.4		
	180°	170 x 120	1.4			1.4		
		140 x100	0.3			0.7		
		100 x 100	0.2			0.6		
		80 x 80	0.1			0.4		
		60 x 60 Anterior maxilla	0.1			0.4		
		60 x 60 Right maxillary molar	0.2			0.1		
		60 x 60 Right mandibular molar	0.2			0.1		
		40 x 40 Anterior maxilla	0.1			0.2		
		40 x 40 Right maxillary molar	0.1			0.0		
		40 x 40 Right mandibular molar	0.3			0.1		
AD Goren (2013) [21]			Using eyeglasses			No eyeglasses		
	-	Full field of view	0.142			0.433		
R. Prins (2011) [20]			Adult female phantom			Adult male phantom		
	-	A full feed of the view	0.133 ± 0.002			0.133 ± 0.001		
			0.051 ± 0.002			0.045 ± 0.001		
E Hofmann (2014) [22]			3D Accuitomo 170	KaVo 3D eXam	Pax Zenith 3D		Pax Reve 3D	Picasso Trio
	-	-	8.30	1.53	2.38		0.54	0.32

Discussion

Although CBCT has been extensively used for the dental management of patients, it is neither monitored nor regulated, and there are no clear guidelines for limiting exposure. The ALARA (as low as reasonably achievable) concept states that the doses should be as reasonably achievable whenever radiograph imaging is taken. In recent years, CBCT imaging has become a common diagnostic tool in endodontics. In the last decade, the number of CBCT-related papers published annually has increased (Fig 1). This has resulted in a large body of literature. A systematic review of the literature related to CBCT imaging of the OMF was performed to evaluate the indications, benefits, and drawbacks of this new image-acquisition technique. However, few studies have quantified the radiation scattered to the eye's lens from the CBCT. A study by Yuan et al. asserts the potential risk to the eye's lens from CT imaging. Since the use of CBCT is more recent and growing, "similar risks can be anticipated in patients undergoing CBCT," especially when the primary beam is closer to that area. The threshold of eye lens damage has decreased from 2 Gy to 0.5 Gy due to the emerging body of evidence [23].

This study aims to assess the radiation dose from a scattered beam to the eye lenses of adult patients using CBCT during diagnostic dental imaging. The current review included six articles that measured the dose to the eye lens using Phantoms. We demonstrated a large variation in the dose received by the eye, which could be attributed to the CBCT type, dosimeter used, scanning parameters, degree of rotation, and FOV used. 

Pauwels et al. studied the effect of the FOV on the effective dose [17]. They found that the mean effective doses at large, medium, and small FOV were 149, 101, and 54 Sv, respectively. The studies by R Pauwels et al. (2014) and Mutalik et al. (2020) took into consideration the degree of rotation of CBCT (180°, 360°) in which a different dose was measured [17,23].

However, using different dosimeters showed no significant difference in the doses reported: TLDs were used in two studies [17,22], and OSLs were used in the other three [20,21,23]. In addition, the use of male and female phantoms did not differ in the study by R. Prins (2011). Most studies reported the left and right eye measures separately, although two studies reported the measures as averages [20,22].

A study presenting measurements on multiple machines [22] presented the average eye dose, whereas separate eye doses were presented in studies with a single CBCT. 

The variation in effective dose correlates to various parameters, including scanners and the scanned region scanned, scanner parameters, size of the FOV, exposure time (s), tube current (mA), and finally, the energy potential (kV) [22,23]. Thus, owing to the inherent risks of radiation, its use should be justified individually to ensure that the benefits to the patient exceed the risks. Scatter Doses of 8 were shown only in studies [17,22,23] using the 3-D Accuitomo 170 machine.

## Conclusions

This review included six articles with actual measurements of the scattered dose to the eye lens and thyroid using an adult phantom. It showed that scattered radiation dose to the eye lens and thyroid during a CBCT scan could exceed 8 mGy. Nevertheless, the dose varied considerably among authors included in the review due to the machine type, many exposure settings (including the FOV and the degree of rotation), and the phantom used. Therefore, strict regulation of patient protection policies should be implemented and audited. In addition, proper justification criteria for ordering CBCT should be assessed, and continuous reviewing of protocols and parameters is also needed; patient doses should also be tracked and reported. Moreover, diagnostic reference levels should be established for benchmarking and optimization in practice.
